# Physical Work Demands of Childcare Workers in Denmark: Device-Based Measurements and Workplace Observations Among 199 Childcare Workers from 16 Day Nurseries

**DOI:** 10.1093/annweh/wxaa041

**Published:** 2020-06-03

**Authors:** Andreas Holtermann, Peter Fjeldstad Hendriksen, Kathrine Greby Schmidt, Malene Jagd Svendsen, Charlotte Diana Nørregaard Rasmussen

**Affiliations:** 1 Department of Musculoskeletal Disorders and Physical Workload, National Research Centre for the Working Environment, Lersø Parkallé 105, Copenhagen Ø, Denmark; 2 Department of Sports Science and Clinical Biomechanics, University of Southern Denmark, Campusvej 55, Odense M, Denmark

**Keywords:** accelerometry, childcare workers, observation, physical work demands

## Abstract

**Objectives:**

Childcare workers in Denmark have high prevalence of musculoskeletal pain (MSP) and sickness absence, but the existing knowledge of their physical work demands is limited, hampering preventive initiatives. This study aimed to assess the physical work demands with accelerometers and workplace observations of childcare workers handling children age 0–3.

**Methods:**

Data collection consisted of an electronic survey, anthropometric measurements, accelerometer measurements providing information of physical activity types and postures with Acti4 software from five consecutive workdays, as well as 4-h visual workplace observation per childcare worker from 16 Danish nurseries.

**Results:**

In total, 199 childcare workers were enrolled in the study. A total of 4181 working hours of accelerometer measurements and 722 h of workplace observations were carried out. Accelerometer measurements showed that they spent about half of the working day (44.8%) in sedentary postures, and the rest standing (22.8%), moving (13.0%), walking (14.6%), running (0.1%), and climbing stairs (0.7%), with 4.1% in knee straining postures (kneeling and squatting) and 4.3% forward trunk inclination >60°. Workplace observations showed that they carried children 1.8% of the working hours.

**Conclusions:**

Physical work demands of Danish childcare workers are characterized by about half of the workday being sedentary, and the remaining of the workday being quite evenly distributed between standing and dynamic activities, with low exposures to carrying children. Their exposure to forward bending of the trunk and knee straining postures could impose a risk for MSP and sickness absence, and preventive initiatives should be considered.

## Introduction

The main aim of childcare work in Danish day nurseries is to provide safe, healthy, and developing environment for children age 0–3 years. On a daily basis, childcare workers perform a variety of tasks meeting the children’s basic needs, e.g. diaper changes, feeding hungry mouths, getting down to eye level, providing comfort, and changing clothes. These work tasks unavoidable involve physical activities such as lifting, carrying, and supporting the children’s weight, as well as requiring the childcare workers to do postures and movements such as bending forward, squatting, kneeling, and sitting on the floor.

High physical work demands increase the risk of musculoskeletal pain (MSP) and sickness absence (Lund *et al.*, 2006; Andersen *et al.*, 2007). For example, high amounts of occupational kneeling and squatting, forward bending of the back, and elevation of the arms increase the risk for MSP (Kumar, 1990; Coenen *et al.*, 2013; Herquelot *et al.*, 2014; Palm *et al.*, 2018; Tammana *et al.*, 2018) and sickness absence (Andersen *et al.*, 2016).

Danish childcare workers report high physical exertion during work, a high prevalence of MSP and sickness absence (Rasmussen *et al.*, 2018). Compared with the national average across all professions in Denmark, childcare workers report higher physical exertion at work and MSP in lower back and neck/shoulders (Tal og fakta om arbejdsmiljøet, 2018). However, surprisingly little research has been conducted on the physical work demands among childcare workers (Labaj *et al.*, 2016; Linnan *et al.*, 2017). The limited existing research is based on device-based measurements and workplace observations of very few childcare workers, or on less reliable self-reported measurements (Gratz and Claffey, 1996; Okuno *et al.*, 1997; Shimaoka *et al.*, 1998; Horng *et al.*, 2008; Labaj *et al.*, 2016). A recent study used accelerometer measurements to investigate the intensity of physical activity of childcare workers per day (Ward *et al.*, 2018), but did not provide information about the amount of work time childcare workers perform specific physical work demands such as forward bending, lifting, carrying, standing, walking, and sitting on the floor. Another study measured these physical work demands by device-based and video measurements, but than on 22 childcare workers for 3.5 h only (Labaj *et al.*, 2016).

Therefore, the aim of this study was to assess the physical work demands of Danish childcare workers handling children age 0–3 based on accelerometer measurements and workplace observations.

## Methods

### Study population

This paper is part of the TOY-project (ISRCTN10928313) which aims to build a greater research-based knowledge regarding physical work demands among childcare workers, and to test whether it is possible to reduce their perceived physical exertion and MSP through a participatory ergonomic intervention. Details regarding the recruitment procedures and inclusion criteria are presented in the protocol paper (Rasmussen *et al.*, 2018).

In short, the data come from baseline measurements and workplace observations from childcare workers at 16-day nurseries handling children aged 0–3 years. Eligible day nurseries were located in the Copenhagen municipality, had children in the age group 0–3 years, and a minimum of nine childcare workers employed. Twenty-nine nurseries would like to participate and were eligible. Resource restrictions limited the number of nurseries in the project to 16 nurseries, which were randomly drawn from the sample of twenty-nine, balanced by size (Rasmussen *et al.*, 2018). Participation in the project was decided at nursery level, but each individual childcare worker decided on participation in the research evaluation of the project. No incentive for participation was given. The childcare workers were informed of the general aims of the study and gave written consent to participate. All procedures were performed according to the declaration of Helsinki. The study was verified by The Danish Ethics Committee to be approved via the National Research Centre for the Working Environments authorization for low risk non-invasive studies on healthy consenting adults, and do not need further reports to the local ethics committee (reference number 16048606). The study is registered in the ISRCTN Registry (ISRCTN10928313). Criteria of exclusion for the childcare workers were severe allergy to band-aid, pregnancy, or fever on the day of the baseline anthropometric measurements.

### Data collection

Data were obtained from mid August to late October in 2017. All data were de-identified and analysed anonymously. The data collection methods comprise questionnaires, anthropometric measurements, accelerometer measurements, and workplace observations. Field data collection occurred concurrently in two nurseries each week. Prior to field data collection, the childcare workers filled out an online questionnaire about their sex, age in years, job title, current seniority, current smoking habit, self-rated general health (Ware *et al.*, 2005), work hours, and perceived physical exertion at work (Borg, 1962).

On Monday or Tuesday, baseline objective physical measures of body height, body weight (kg), body mass index [body weight/(body height^2^) (kg m^−2^)], and blood pressure (mmHg) (seated with flexed elbow at heart level) were performed and accelerometers were attached. Five AX3 accelerometers (3-Axis Logging Accelerometer; Axivity Ltd, Newcastle upon Tyne, UK) were attached on the childcare worker’s skin with adhesive tape (Hair-Set double-sided adhesive tape; 3M Company, Maplewood, MN, USA) and secured with transparent adhesive film (OPSITE FLEXIFIX; Smith & Nephew plc, London, UK) for five workdays. The accelerometers were mounted at the following positions: (i) the trunk, on the spine just below the processus spinosus at the level of T1–T2, (ii) the dominant arm, laterally and 3 cm distal to the deltoid insertion, (iii) the right thigh at the most muscular part of the quadriceps femoris, midway on the line between the anterior inferior iliac spine and the top of the patella, and (iv) the right calf and the left calf on the flat part of the soleus and gastrocnemius aponeurosis just distal to the lateral and medial heads of the gastrocnemius. In a paper diary, childcare workers recorded what time they ‘woke up’, ‘arrived at work’, ‘left from work’, ‘went to sleep’ and/or if any of the devices were detached in a diary for all days of accelerometer measurements.

At some point during the week of measurements, the childcare worker was observed by a rater. A modified Task Recording and Analysis on Computer (TRAC) and portable ergonomic observation approach (POE) (Fransson-Hall *et al.*, 1995; Monique and Frings-Dresen, 1995) were used to develop the observational tool called TRACK. The development of TRACK was based on another observation tool developed for eldercare workers (Karstad *et al.*, 2018). Items in the TRACK instrument are physical work demands we are unable to assess by accelerometer measurements (e.g. lifting, carrying, and sitting on the floor). The TRACK instrument has been shown to have an overall high inter-rater reliability (Svendsen *et al.*, 2020). Observations were conducted with 2/3 as morning (8.00–12.00) and 1/3 as afternoon (13.00–17.00) sessions to cover the variance of childcare work best. From 12.00 to 13.00 most children sleep and childcare workers have lunch breaks and meetings. After lunch, the number of children attending nursery decreases and as a result, the majority of working hours for the childcare workers is scheduled for earlier parts of the days. To minimize disturbance in the classroom/ward, only one childcare worker was observed at a time. The workplace observations took place using a handheld computer (GT-P3100 or SM-T280; Samsung, Suwon, South Korea) with Pocket Observer software (Pocket Observer version 3.3.46; Noldus Information Technology, Wageningen, The Netherlands) able to record start-stop or occurrence of observed items from the TRACK-tool. The TRACK instrument, rater training, and observation procedures are described in Svendsen *et al.* (2020).

### Data processing

Questionnaire data were exported to an Excel spreadsheet (Excel 2010; Microsoft Corporation, Albuquerque, USA) upon answering. If the childcare workers had missing data, they were contacted after 2 weeks and 1 month via text messages and phone calls to fill in missing data fields.

Anthropometric data were carefully entered in an Excel spreadsheet, and subsequently double-checked for correctness.

The manufacturer’s software (OMGUI Version 1.0.0.30; Axivity Ltd, Newcastle upon Tyne, UK) was used for initialization of accelerometers at a sampling rate of 25 Hz and data download. For further analyses, a custom-made MatLab-based software (Acti4; The National Research Centre for the Working Environment, Copenhagen, Denmark) was used to determine physical work demands (e.g. sitting, standing, moving, walking, running, and stair climbing) (Skotte *et al.*, 2014), as well as arm elevation and forward bending (Korshoj *et al.*, 2014), and the knee straining postures squatting and kneeling (Hendriksen *et al.*, 2020). The Acti4 software is described elsewhere, and shown to estimate these physical work demands with high sensitivity and specificity (Skotte *et al.*, 2014).

The diaries were digitized and used to differentiate the accelerometer measurements into ‘working hours’, ‘leisure time’, and ‘sleep’ in the Acti4 software. Only accelerometer measurements from ‘working hours’ (subsequently in this paragraph, referenced as ‘workday(s)’) was used for the analysis of the occupational physical work demands. Then valid workdays for each subject were determined. The criteria for a valid workday were ≥4 h of accelerometer measurements, which were in line with previous studies (Jorgensen *et al.*, 2019). A criterion of ≥3 h was considered due to a large subpopulation of childcare workers having less than 37 h week^−1^ of work (28–31 h week^−1^) compared with previously studied groups. This was rejected as this would primarily include the days when the accelerometers were attached or detached from the subject, which were seen as high-risk days for biased behaviour. The ≥4 h criteria primarily included full working days. Due to different amounts of valid workdays between subjects, the dataset was normalized to an average working day for each subject. Thus, for each subject each unique accelerometer derived variable was aggregated into a new dataset as a single mean of the valid workday(s) for each subject.

The Pocket Observer recordings were processed using The Observer XT software (The Observer XT version 14; Noldus Information Technology, Wageningen, The Netherlands) to derive duration and frequency of observed physical work demands.

### Statistical analysis

All processed data were imported into SPSS (IBM SPSS Statistics for Windows, Version 24.0, IBM Corp, Armonk, NY) and derived variables (percentage of work hours, occurrence per hour) were first calculated for each childcare worker and subsequently the percentile descriptive data were generated from eligible childcare workers for each variable.

## Results

**Figure 1.**
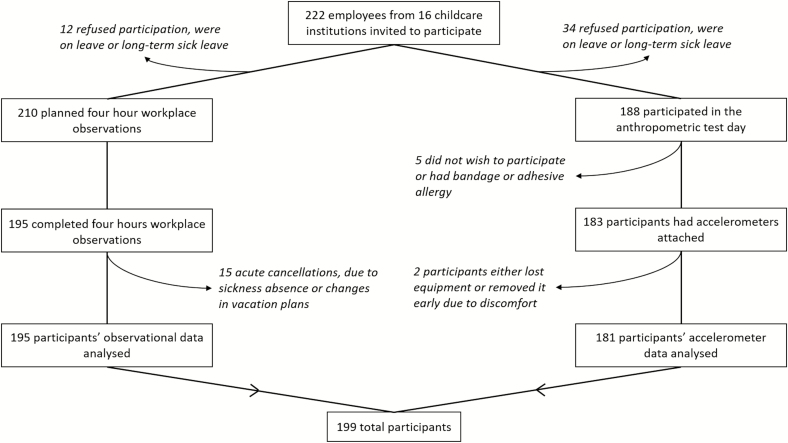
Participant flow for workplace observations and accelerometer measurements.

Fig. 1 shows the flowchart of the study. A total of 222 employees from 16 Danish childcare nurseries were invited and consented to participation in the TOY-project. Of the 222 eligible childcare workers, workplace observations were performed and data analysed on 195 participants. Accelerometers were attached on 183 participants and 181 participants had accelerometer data for analyses. The missing data were due to refused participation, sick leave, acute cancellations, lost or removed equipment, or discomfort. In total, data from 199 childcare workers were included in the project (Fig. 1).

Demographics and health characteristics of the 199 childcare workers enrolled in the study are presented in Table 1. The childcare workers were predominantly females (86.4%), on average 37 years old, having a body mass index of 25.3, and an average blood pressure of 118.1 systolic mmHg and 78.9 diastolic mmHg. Moreover, 23.6% were current smokers.

**Table 1.** Demographic and health characteristics of the childcare workers (*n* = 199). Values are numbers, percentage, mean, and/or SDs.

Variables	*N*	%	Mean (SD)
Sex (*n* = 199)			
Female	172	86.4	
Male	27	13.6	
Age (*n* = 199)			
In years			37
BMI^a^ (kg m^−2^)	189		25.3 (5.4)
Blood pressure (mmHg)			
Systolic	187		118.1 (15.1)
Diastolic	187		78.9 (10.4)
Current smoking (*n* = 189)			
Yes	47	23.6	
No	142	71.4	
Working hours (*n* = 193)			
Hours per week			35
Job title (*n* = 199)			
Childcare worker	115	57.8	
Assistant childcare worker	72	36.2	
Temporary hire or intern	12	6	
Seniority in current job (*n* = 192)			
<3 months	21	10.9	
3 to <12 months	32	16.7	
12 to <60 months	55	28.6	
60 to <120 months	42	21.9	
≥120 months	42	21.9	
Self-reported overall health evaluation (*n* = 189)			
In very good health	49	25.9	
In good health	81	42.9	
In average health	55	29.1	
In bad health	4	2.1	
Self-rated physical exertion at work (0–10^b^)	178		5.9 (1.8)

SD, standard deviation.

^*a*^Body mass index.

^*b*^0 = effortless, 10 = hardest possible.

On average, the childcare workers worked 34.9 h weekly and the majority (57.8%) were childcare workers, 36.2% assistant childcare worker, and 6% had a temporary hire or were an intern. The largest proportion of the childcare workers (28.6%) had a seniority of 12–60 months in their current job. The majority (68.8%) stated having either a good (42.9%) or a very good health (25.9%). The mean self-rated physical exertion at work was 5.9 on a 0–10 scale (0 = effortless, 10 = hardest possible).

The accelerometer-based physical work demands are presented in Table 2. A total of 4181 working hours of accelerometer measurements were carried out on 181 eligible childcare workers. On average, 3.6 workdays were measured per childcare worker, with an average duration of 6.5 working hours per day. Additionally, a total of 722 h of workplace observations were carried out on 195 eligible childcare workers with an average duration of 3.7 h per observation. Of the 195 observations, 132 (68%) were conducted as morning and 63 (32%) as afternoon sessions.

**Table 2.** Physical work demands during working hours measured by accelerometers and workplace observations presented as percentage of work hours (%) or frequency per work hour (*n* per hour). Values are mean and SDs.

Variable	*N*	Mean	SD
Accelerometer measurements			
Kneeling (%)	175	2.5	2.8
Squatting (%)	175	1.6	1.1
Lie (%)	181	1.8	2.2
Sit (%)	181	43.0	8.8
Stand (%)	181	22.8	5.9
Moving^a^ (%)	181	13.0	3.4
Walking (%)	181	14.6	3.7
Running (%)	181	0.1	0.1
Stairs (%)	181	0.7	0.4
Cycling (%)	181	0.2	0.4
Arms elevated over 60° (%)	180	5.0	3.1
Arms elevated over 90° (%)	180	1.1	0.9
Forward trunk inclination above 30° while on feet (%)	179	10.6	4.1
Forward trunk inclination above 60° while on feet (%)	179	4.3	1.9
Steps (*n* per hour)	181	1018	254
Workplace observations			
Carrying, total^a^ (%)	195	2.6	2.8
Carrying, total (*n* per hour)	195	2.5	2.1
Carrying, child (%)	195	1.8	2.2
Carrying, child (*n* per hour)	195	1.7	1.7
Manual handling^b^, child (*n* per hour)	195	8.2	4.6
Manual handling^b^, total^c^ (*n* per hour)	195	12.4	6.8
Sitting, on floor (%)	195	12.4	9.0
Sitting, on floor (*n* per hour)	195	2.0	1.4

^*a*^Time on feet not identified as standing or walking.

^*b*^Child or object moved or supported while having contact to the surface.

^*c*^Children and objects merged.

On average, the primary part of the working hours (51.3%) were spent by the childcare workers in physical activities such as standing (22.8%), moving (13.0%), walking (14.6%), running (0.1%), and climbing stairs (0.7%). About half of the working hours (44.8%) were spent sedentary, including sitting (43.0%) or lying (1.8%). Of this time spent sedentary, the workplace observations showed that the childcare workers were sitting on the floor in 12.4% of the working hours.

Moreover, 4.1% of the time measured from the 175 eligible childcare workers were used in the knee straining postures, kneeling (2.5%) and squatting (1.6%). Furthermore, the accelerometer measurement showed that the childcare workers had their arms elevated >60° and >90° in 5 and 1.1% of the working hours, respectively. Moreover, the 179 eligible childcare workers had a forward trunk inclination >30° and >60° in 10.6 and 4.8% of the working hours, respectively.

On average, the childcare workers carried children or objects in 2.6% of the working time, on average 2.5 times per hour. The childcare workers carried children 1.7% of the working hours, on average 1.7 times per hour. Moreover, the childcare workers were handling children or objects (while the child/object has contact to the surface) in 8.2% of the workday, corresponding to 12.6 times per hour.

Percentage childcare workers performing particularly demanding physical work demands in relatively long time of the workday (≥10%) are presented in Table 3. Particularly, 7.4% of the childcare workers performed the knee straining postures (squatting and kneeling) ≥10% of the workday, and as much as about half (51.4%) of the childcare workers performed forward trunk inclination >30° while on feet ≥10% of the workday. The prevalence performing the other demanding physical work demands ≥10% of the workday was low.

**Table 3.** Percentage of childcare workers performing selected physical work demands ≥10% of the workday, measured by accelerometers or workplace observations.

Physical work demands	*N*	% of *N*
		≥10% of the workday
Accelerometer measurements		
Kneeling	175	3.4
Squatting	175	0.0
Knee straining^a^	175	7.4
Arms elevated >60°	180	1.7
Arms elevated >90°	180	0.6
Forward trunk inclination >30° while on feet	179	51.4
Forward trunk inclination >60° while on feet	179	1.7
Workplace observations		
Carrying, child	195	1.0
Carrying, total^b^	195	2.6

^*a*^Kneeling and squatting merged.

^*b*^Children and objects merged.

## Discussion

To our knowledge, this is the largest study of childcare workers with accelerometer measurements over several consecutive workdays and workplace observations of physical work demands. Of the 199 childcare workers included in the study, a total of 4181 h of accelerometer measurements over an average of 3.6 workdays on 181 eligible childcare workers and 722 h of workplace observations with an average of 3.7 h for 195 eligible childcare workers were carried out. The presented findings contain information of the physical works demands among childcare workers going far beyond the existing research literature. Thus, we consider the findings from this study valuable for improving our knowledge about the need for, and where to target, preventive workplace interventions among childcare workers.

Based on the accelerometer measurements over several workdays, we found that the childcare workers spent about half of the workday (44.8%) sedentary (i.e. sitting and lying). Compared with similar accelerometer measurements and analyses of sedentary behaviour in other job groups, the childcare workers spend more of their working hours sedentary than cleaners and manufacturing workers (Jorgensen *et al.*, 2019) and nurses (Loef *et al.*, 2018). However, the childcare workers spent less of their workday sedentary than workers in transportation and office/administration workers (Gilson *et al.*, 2019; Jorgensen *et al.*, 2019). We consider the exposure to sedentary behaviour of the childcare workers to be of minor concern for imposing health impairments compared with occupational groups which might spend too much occupational time sedentary (e.g. long haul drivers) or occupational groups which might spend too little time sedentary for having sufficient rest and recovery over the workday (e.g. cleaners). The workplace observations showed that the childcare workers spent quite much of their sedentary time by sitting on the floor (i.e. 27% of their sedentary time and 12% of workday). We are not aware of prospective epidemiological studies investigating the dose–response relationship between exposure to work time sitting on the floor and health impairments. Thus, we do not know if this exposure to sitting on the floor is of concern for health issues for the childcare workers, like low back pain. This ought to be investigated in with device-based measurements during normal work in prospective study designs.

The childcare workers spent about 23% standing and 28% in more dynamic activities (13% moving and 15% walking). The exposure to these work demands are of shorter duration than seen in other occupational groups with comparable accelerometer measurements, such as cleaners (standing 26% and walking 45%) and manufacturers (standing 38% and walking 34%). Considering that the childcare workers are spending about half of the workday sedentary, and the quite even distribution between standing and dynamic activities, we do not consider them to impose a risk for health impairments, such as low back pain among the childcare workers.

The percent working time spent on physical activity demands with higher intensity like stair climbing and running (<1%) is of too low duration to be likely to influence the health of the childcare workers. Our finding of a low duration of physical activity of higher intensity at work among childcare workers is in line with the previous accelerometer study among childcare workers (Ward *et al.*, 2018), and supports that physical activities of higher intensity during productive work (e.g. by being role models for facilitating physical activity for the children) could be considered to be promoted for improving the fitness and health of the childcare workers (Ward *et al.*, 2018; Holtermann *et al.*, 2019).

Based on the workplace observations, the childcare workers carried children 1.7% of the working hours, on average 1.7 times per hour, which corresponds to 1.02 min per hour and an average duration of 36 s. Unfortunately, we were not permitted to measure the weight of the children, and we found it difficult to develop reliable estimates by the observer of the weight of the children. Based on the Danish Health Authority general growth rates for boys and girls, the mean weight curves for boys are: 1 year old’s: 10 kg; 2 years: 12 kg; 3 years: 15 kg, and for girls: 1 year: 10 kg; 2 years: 12 kg; 3 years: 14 kg. However, based on the current research literature (Coenen *et al.*, 2014), we generally consider this exposure to carrying of children to be too low to be likely to significantly increase the risk for low back pain and sickness absence among the childcare workers. This low exposure to carrying of children is likely a result of years with preventive initiatives for reducing carrying of children in day nurseries in Denmark (e.g. campaigns titled ‘I can—I want to!’ (Arbejdstilsynet og BrancheFællesskabet for Arbejdsmiljø for Velfærd og Offentlig administration, 2020) and ‘Let the child do it!’ (Svith and Steven, 2007), and might not apply to childcare work in other countries. Moreover, the childcare workers were manually handling children 8.2 times per hour and objects 4.4 times per hour (i.e. lifting, pushing/pulling, or otherwise ‘assisting’ the child/object while being in contact with the ground surface). Since the child is in contact with the ground surface during two out of three types of manual handling included, these will impose a relatively low biomechanical load on the childcare worker. Additionally, the frequency of manual handlings allows for roughly 7 min and 19 s restitution between events on average. Thus, the amount of observed manual handling is not likely a risk for MSP or sickness absence for the childcare workers.

The accelerometer measurements on the upper arm showed that the childcare workers spent about 5% of the workday with the arm elevated >60°. This exposure is comparable with the exposure to arm elevation during the workday among cleaners (6–7%), while being considerably lower than among occupational groups like different types of construction work (e.g. machine operators, paver, finishing of products) having between 10 and 12% of the workday with the arm elevated >60° (Palm *et al.*, 2018). Extensive working time with elevated arms is reported to increase the risk for shoulder pain and disorders (van Rijn *et al.*, 2010), but the prospective dose–response relationship between individual-level duration of arm elevation and shoulder pain and sickness absence based on device-based measurements during normal workdays is unknown. More than 10% of the workday with unsupported arm elevation >60° is suggested to imply an increased risk for shoulder disorders (Hansson *et al.*, 2016). However, because of the childcare workers relatively short duration of arm elevation, and their low exposure to carrying of children and manual handling, arm elevation is not likely to increase their risk for neck/shoulder pain.

The exposure to forward trunk inclination >60° while on feet was 4.3% of the workday among the childcare workers. This exposure to forward trunk inclination is comparable to what have previously been found with the same measurement methodology among cleaners (4.6% of workday), while being a little higher than for manufacturing workers (3.1%) (Jorgensen et al., 2019). About half (51.4%) of the childcare workers performed forward trunk inclination >30° while being on their feet more than 10% of the workday. Occupational exposure to forward bending of the back is an acknowledged risk factor for low back pain and sickness absence (Coenen *et al.*, 2013; Andersen *et al.*, 2016). However, these studies have been based on self-reported measures of forward trunk inclination, which can be imprecise and potentially biased. Thus, we cannot make an evidence-based conclusion on the risk for low back pain from forward trunk inclination for this population. However, based on the existing knowledge from studies based on self-reported forward inclination, minimizing forward trunk inclination for childcare workers would be the cautionary advice.

The accelerometer measurements of the knee straining postures, kneeling and squatting, revealed that the childcare workers spent 2.5% of the workday kneeling and 1.6% squatting. Moreover, 7.4% performed the knee straining postures (squatting and kneeling) more than 10% of the workday. Occupational exposure to kneeling and squatting is well known to increase risk for knee pain and disorders (Herquelot *et al.*, 2014). However, we are not aware of previous studies investigating exposure to these knee straining postures during natural working environments using device-based measurements over full working days. Thus, future studies on exposure measurements of knee straining postures over several working days with accelerometers are recommended to improve our knowledge about exposure levels of knee straining postures, risk for knee disorders and improved preventive workplace interventions. Due to the lack of an established device-based dose–response relationship between occupational kneeling and squatting with knee pain and disorders, no conclusion can be made on the relevant risk for this population based on our measurements. However, minimizing exposure to kneeling and squatting for childcare workers would be the cautionary advice due to the established relationship from self-report studies.

Overall, the childcare workers reported high self-rated physical exertion at work (average of 5.9 on a 0–0 scale, 0 = effortless; 10 = hardest possible). However, based on the accelerometer measurements and workplace observations, the physical work demands of the childcare workers were characterized by almost half of the workday being sedentary, and the remaining of the workday being quite evenly distributed between moving, standing, and walking, with low exposure to carrying and more intensive activities like running and stair climbing. Overall, these exposures are lower than we have found with similar accelerometer measurements (except for sedentary time being higher) among cleaners and manufacturing workers (Jorgensen *et al.*, 2019). Thus, based on all exposure measurements we consider the physical work demands among the childcare workers to be relatively low and thus not constituting a significant increased risk for impaired health. We see this discrepancy between the high reported physical exertion and relatively low physical work demands as an ‘*ergonomic paradox*’, which ought to be further investigated to understand the underlying causes and mechanisms to this discrepancy.

However, we also found exposures to forward bending of the trunk and knee straining postures which could impose a risk for MSP and sickness absence. Thus, we think that day nurseries ought to consider implementation of cost-effective interventions to reduce the exposure to forward bending of the back and knee straining postures of childcare workers. This could, for example, be by designing the cribs and changing stations so the children can manage, following appropriate training, to climb up themselves.

### Strength and limitations

We acknowledge some strengths and limitations of this study. One of the major strengths is its considerable contribution to what is currently a very limited scientific literature regarding childcare workers’ physical work demands. We used validated accelerometer measurements and software (Skotte *et al.*, 2014) to capture the physical activity demands and postures of the childcare workers over several working days. Moreover, as recommended by Takala *et al.* (2010), we used multiple tools to capture different aspects of the childcare workers’ work, which enable us to make a detailed picture of the childcare workers’ physical activities and workloads. The workplace observations using the reliable TRACK instrument captured several of the physical work demands not measured with the accelerometers (e.g. sitting on floor and carrying) (Svendsen *et al.*, 2020).

A potential weakness of the study is selection bias, in which 34 out of the 222 childcare workers enrolled in this study were not participating in measurements. It is plausible that the childcare workers who were willing to participate may be healthier than the ones who did not participate. However, the proportion of the workers not willing to participate is so low, that we consider it not likely to have a significant influence on the main results of this study. Another limitation is that the participating day nurseries were only recruited from the Copenhagen municipality, which might not be representative for all of Denmark. However, we have no reason to believe that the physical work demands, and organization of work is different in the Copenhagen municipality compared with other Danish municipalities.

## Conclusion

Based on the accelerometer measurements and workplace observations, the physical work demands of the childcare workers were characterized by almost half of the workday being sedentary, and the remaining of the workday being quite evenly distributed between standing and dynamic activities, with low exposures to carrying and activities with high intensity like running and stair climbing. Overall, these exposures are lower than for other occupational groups we have comparable data on, like cleaners and manufacturing workers. Thus, we overall consider the physical work demands among the childcare workers to be relatively low and not increasing their risk for health impairments. However, we also found exposures to forward bending of the trunk and knee straining postures which could impose a risk for MSP and sickness absence, and preventive initiatives should be considered. Because of the high rates of MSP and sickness absence of the childcare workers, and the limited research-based knowledge on their exposure to physical work demands with accelerometer measurements and workplace observations, further research are needed to inform better preventive workplace interventions for childcare workers.

## Funding

The study is externally funded by The Danish Working Environment Research Fund (grant no. 2-2016-03 20165101186). We would also like to acknowledge Klaus Hansen, Dorte Ekner, Jørgen Skotte, Pernille Kold Munch, and the TOY-projects student helper team for their effort during the data collection.

## Authors’ contributions

AHO, PFH, and CNR participated in the discussion and drafting of the conceptual design of the study and wrote the initial protocol as well as the application for funding. PFH, KGS, MAS, CNR, and AHO participated in finalizing the measurement and observational protocols. MAS was responsible for planning and organizing the team carrying out data collection. KGS was part of the data collection team. PFH started drafting the paper and AHO wrote the manuscript. All authors have read and commented on the draft version as well as approved the final version of the manuscript.

## Competing interests

The authors declare that they have no competing interests.

## References

[CIT0001] Andersen LL, Fallentin N, Thorsen SV et al (2016) Physical workload and risk of long-term sickness absence in the general working population and among blue-collar workers: prospective cohort study with register follow-up. Occup Environ Med; 73: 246–53. 26740688 10.1136/oemed-2015-103314

[CIT0002] Andersen JH, Haahr JP, Frost P (2007) Risk factors for more severe regional musculoskeletal symptoms: a two-year prospective study of a general working population. Arthritis Rheum; 56: 1355–64. 17393441 10.1002/art.22513

[CIT0003] Arbejdstilsynet and BrancheFællesskabet for Arbejdsmiljø for Velfærd og Offentlig administration I can I want to. Information for parents. Available at https://at.dk/media/5044/kanselvvilselv-pjece-uk-v15.pdf. Accessed 27 March 2020.

[CIT0004] Borg G (1962) Physical performance and perceived exertion. Lund: Berlingska boktryckeriet, C. W. K. Gleerup; Copenhagen: E. Munksgaard.

[CIT0005] Coenen P, Gouttebarge V, van der Burght AS et al (2014) The effect of lifting during work on low back pain: a health impact assessment based on a meta-analysis. Occup Environ Med; 71: 871–7. 25165395 10.1136/oemed-2014-102346

[CIT0006] Coenen P, Kingma I, Boot CR et al (2013) Cumulative low back load at work as a risk factor of low back pain: a prospective cohort study. J Occup Rehabil; 23: 11–8. 22718286 10.1007/s10926-012-9375-z PMC3563950

[CIT0007] Fransson-Hall C, Gloria R, Kilbom A et al; Stockholm Music 1 Study Group. (1995) A portable ergonomic observation method (PEO) for computerized on-line recording of postures and manual handling. Appl Ergon; 26: 93–100. 15677005 10.1016/0003-6870(95)00003-u

[CIT0008] Gilson ND, Hall C, Holtermann A, et al (2019) Sedentary and physical activity behavior in “Blue-Collar” workers: a systematic review of accelerometer studies. J Phys Act Health: 1–10. 10.1123/jpah.2018-0607 31469366

[CIT0009] Gratz RR, Claffey A (1996) Adult health in child care: health status, behaviors, and concerns of teachers, directors, and family child care providers. Early Child Res Q; 11: 243–267.

[CIT0010] Hansson G, Arvidsson I, Nordander C (2016) Riktvärden för att bedöma risken för belastningsskador, baserade på tekniska mätningar av exponering. Lund, Sverige: Arbets- och miljömedicin.

[CIT0010a] Hendriksen PF, Korshøj M, Skotte J et al. (2020) Detection of kneeling and squatting during work using wireless triaxial accelerometers. Ergonomics; 5: 1–11. 10.1080/00140139.2020.1734668 32100646

[CIT0011] Herquelot E, Bodin J, Petit A et al (2014) Long-term persistence of knee pain and occupational exposure in two large prospective cohorts of workers. BMC Musculoskelet Disord; 15: 411. 25475051 10.1186/1471-2474-15-411 PMC4289228

[CIT0012] Holtermann A, Mathiassen SE, Straker L (2019) Promoting health and physical capacity during productive work: the Goldilocks Principle. Scand J Work Environ Health; 45: 90–7. 30021029 10.5271/sjweh.3754

[CIT0013] Horng YS, Hsieh SF, Wu HC et al (2008) Work-related musculoskeletal disorders of the workers in a Child Care institution. Arch Phys Med Rehabil; 36: 15–21.

[CIT0014] Jørgensen MB, Gupta N, Korshøj M et al (2019) The DPhacto cohort: an overview of technically measured physical activity at work and leisure in blue-collar sectors for practitioners and researchers. Appl Ergon; 77: 29–39. 30832776 10.1016/j.apergo.2019.01.003

[CIT0015] Karstad K, Rugulies R, Skotte J et al (2018) Inter-rater reliability of direct observations of the physical and psychosocial working conditions in eldercare: an evaluation in the DOSES project. Appl Ergon; 69: 93–103. 29477334 10.1016/j.apergo.2018.01.004

[CIT0016] Korshøj M, Skotte JH, Christiansen CS et al (2014) Validity of the Acti4 software using ActiGraph GT3X+accelerometer for recording of arm and upper body inclination in simulated work tasks. Ergonomics; 57: 247–53. 24392673 10.1080/00140139.2013.869358

[CIT0017] Kumar S (1990) Cumulative load as a risk factor for back pain. Spine (Phila Pa 1976); 15: 1311–6. 2149209 10.1097/00007632-199012000-00014

[CIT0018] Labaj A, Diesbourg T, Dums G, et al (2016) Posture and lifting exposures for daycare workers. Int J Ind Ergon; 54: 83–92.

[CIT0019] Linnan L, Arandia G, Bateman LA et al (2017) The health and working conditions of women employed in child care. Int J Environ Res Public Health; 14. 10.3390/ijerph14030283 PMC5369119 28282940

[CIT0020] Loef B, van der Beek AJ, Holtermann A et al (2018) Objectively measured physical activity of hospital shift workers. Scand J Work Environ Health; 44: 265–73. 29355291 10.5271/sjweh.3709

[CIT0021] Lund T, Labriola M, Christensen KB et al (2006) Physical work environment risk factors for long term sickness absence: prospective findings among a cohort of 5357 employees in Denmark. BMJ; 332: 449–52. 16446280 10.1136/bmj.38731.622975.3A PMC1382535

[CIT0022] Monique HW, Frings-Dresen PPFMK (1995) The TRAC-system: an observation method for analysing work demands at the workplace. Saf Sci; 21: 163–5.

[CIT0023] Okuno M, Uketa S, Nakaseko M et al (1997) Work and workload of nursing personnel in a nursery school and two institutions for handicapped children. Ind Health; 35: 202–11. 9127552 10.2486/indhealth.35.202

[CIT0024] Palm P, Gupta N, Forsman M et al (2018) Exposure to upper arm elevation during work compared to leisure among 12 different occupations measured with triaxial accelerometers. Ann Work Expo Health; 62: 689–98. 29945157 10.1093/annweh/wxy037 PMC6037214

[CIT0025] Rasmussen CDN, Hendriksen PR, Svendsen MJ et al (2018) Improving work for the body—a participatory ergonomic intervention aiming at reducing physical exertion and musculoskeletal pain among childcare workers (the TOY-project): study protocol for a wait-list cluster-randomized controlled trial. Trials; 19: 411. 30064464 10.1186/s13063-018-2788-z PMC6069746

[CIT0026] Shimaoka M, Hiruta S, Ono Y et al (1998) A comparative study of physical work load in Japanese and Swedish nursery school teachers. Eur J Appl Physiol Occup Physiol; 77: 10–8. 9459515 10.1007/s004210050293

[CIT0027] Skotte J, Korshøj M, Kristiansen J et al (2014) Detection of physical activity types using triaxial accelerometers. J Phys Act Health; 11: 76–84. 23249722 10.1123/jpah.2011-0347

[CIT0028] Svendsen MJ, Hendriksen PF, Schmidt KG et al (2020) Inter-rater reliability of ergonomic work demands for childcare workers using the observation instrument TRACK. Int J Environ Res Public Health; 17. 10.3390/ijerph17051607 PMC7084378 32131510

[CIT0029] Svith S, Steven L (2007) Lad dog barnet! Available at https://www.arbejdsmiljoweb.dk/laddogbarnet. Accessed 27 March 2020.

[CIT0030] Takala EP, Pehkonen I, Forsman M et al (2010) Systematic evaluation of observational methods assessing biomechanical exposures at work. Scand J Work Environ Health; 36: 3–24. 19953213 10.5271/sjweh.2876

[CIT0031] Tal og fakta om arbejdsmiljøet. (2018) Book Tal og fakta om arbejdsmiljøet. København, Danmark: Det Nationale Forskningscenter for Arbejdsmiljø.

[CIT0032] Tammana A, McKay C, Cain SM et al (2018) Load-embedded inertial measurement unit reveals lifting performance. Appl Ergon; 70: 68–76. 29866328 10.1016/j.apergo.2018.01.014

[CIT0033] van Rijn RM, Huisstede BM, Koes BW et al (2010) Associations between work-related factors and specific disorders of the shoulder–a systematic review of the literature. Scand J Work Environ Health; 36: 189–201. 20094690 10.5271/sjweh.2895

[CIT0034] Ward DS, Vaughn AE, Hales D et al (2018) Workplace health and safety intervention for child care staff: rationale, design, and baseline results from the CARE cluster randomized control trial. Contemp Clin Trials; 68: 116–26. 29501740 10.1016/j.cct.2018.02.018 PMC5944351

[CIT0035] Ware JE, Kosinski M, Gandek B (2005) SF-36 health survey: manual and interpretation guide. Lincoln, RI: Quality Metric Inc.

